# Radiomics to predict tumor response to combination chemoradiotherapy in squamous cell carcinoma of the anal canal: a preliminary investigation

**DOI:** 10.1186/s41747-025-00559-0

**Published:** 2025-03-22

**Authors:** Andrea Vanzulli, Lucilla Violetta Sciacqua, Filippo Patti, Roza Drebot, Eros Montin, Riccardo Lattanzi, Laura Anna Maria Lozza, Sergio Villa, Davide Scaramuzza

**Affiliations:** 1 Diagnostic and Interventional Radiology Residency Program, Università degli Studi di Milano, Milan, Italy; 2 Department of Diagnostic and Interventional Radiology, Fondazione IRCCS Istituto Nazionale dei Tumori, Milan, Italy; 3 Department of Biomedical Sciences, Humanitas University, Pieve Emanuele (Milan), Italy; 4 Department of Radiation Oncology, Fondazione IRCCS Istituto Nazionale dei Tumori, Milan, Italy; 5 Department of Oncology and Haemato-Oncology, Università degli Studi di Milano, Milan, Italy; 6 Bernard and Irene Schwartz Center for Biomedical Imaging, Department of Radiology, New York University Grossman School of Medicine, New York, NY USA; 7 Center for Advanced Imaging Innovation and Research (CAI2R), Department of Radiology, New York University Grossman School of Medicine, New York, NY USA

**Keywords:** Anal canal, Carcinoma (squamous cell), Magnetic resonance imaging, Precision medicine, Radiomics

## Abstract

**Background:**

Upfront combination chemoradiotherapy (CRT) represents the standard of care for patients affected by stage III squamous cell carcinoma (SCC) of the anal canal, achieving satisfactory results both in terms of overall survival and local disease control. However, a non-negligible fraction of patients obtain incomplete responses, highlighting the need for innovative prognostic tools. We report the preliminary results of a customized radiomic algorithm designed to predict tumor response to CRT in patients affected by SCC of the anal canal.

**Methods:**

We manually annotated pretreatment T2-weighted turbo spin-echo images of 26 consecutive patients with stage III SCC of the anal canal treated with CRT at our institution from 2012 to 2022. Each patient was classified as complete response (CR, 17 patients), or non-complete response (non-CR, 9 patients) based on the absence or presence of residual disease at imaging and endoscopy after treatment. A total of 132 three-dimensional radiomic features were extracted for each patient and fed to a dedicated machine-learning classifier.

**Results:**

Models trained with gray-level co-occurrence matrix features achieved the best performances (accuracy 0.846 ± 0.064, sensitivity 0.900 ± 0.122, specificity 0.833 ± 0.175, area under receiver operating characteristics curve 0.867 ± 0.055), highlighting a more homogeneous distribution of voxel intensities and lower spatial complexity in non-CR patients.

**Conclusion:**

Our radiomic tool accurately predicted tumor response to CRT in patients with stage III SCC of the anal canal, highlighting a more homogeneous tissue composition in poor responders.

**Relevance statement:**

The more homogeneous radiomic texture observed in non-CR patients may be imputable to a dominant neoplastic clone with a relatively low mitotic index (therefore, limited tissue necrosis), intrinsically more resistant to CRT than faster-proliferating tumors.

**Key Point:**

- A non-negligible fraction of patients with anal SCC respond unsatisfactorily to CRT.
- Our radiomic model predicted response to CRT based on pretreatment MRI.
- We observed a more homogeneous tissue composition in poor responders.
- The slow proliferation of a dominant clone may explain non-CR to CRT.

**Graphical Abstract:**

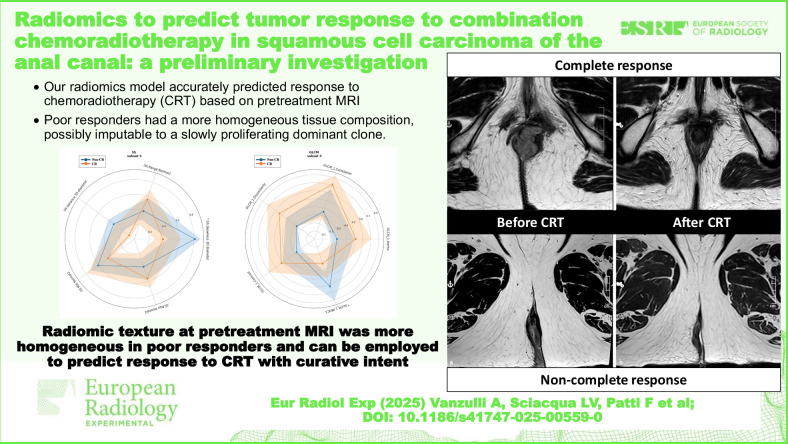

## Background

Malignancies of the anal canal are rare, with 79,091 patients, 9,760 new estimated diagnoses (0.5% of all new cancer cases), and 1,870 estimated deaths in 2023 in the United States alone (0.3% of all cancer deaths) [1, 2]. The cumulative incidence of anal cancer is 1.9 per 100,000 per year (1.6 for men and 2.3 for women, respectively), with a 5-year relative survival (2013–2019) of 70%, ranging from 36.2% to 83.7% based on tumor stage at diagnosis [1, 2]. Approximately 0.2% of the general population will be diagnosed with anal cancer at some point during their lifetime [1]. Squamous cell carcinoma (SCC) is the most prevalent histotype, with human papillomavirus infection being responsible for approximately 90% of all cases, largely imputable to a few selected high-risk genotypes [2–6].

Until the 1980s, the cornerstone of the treatment of SCC of the anal canal was abdominal-perineal resection, resulting in more than 50% of local recurrences, a 5-year overall survival ranging between 40% and 70%, and a marked reduction in quality of life [4]. Nowadays, combination chemoradiotherapy (CRT) has largely replaced demolitive surgery, mostly employed as salvage treatment in selected patients with refractory or relapsing disease [7–11]. Although current treatments achieve overall satisfactory results, a non-negligible percentage of patients only achieve partial responses [1, 12].

The genetic and molecular bases behind primary refractoriness to CRT have not been elucidated yet, and the lack of reliable clinical and molecular predictors paves the way for more advanced approaches, such as radiomics. Radiomics relies on the extraction of high-dimensional data from biomedical images, leveraging the hypothesis that they contain meaningful information regarding tumor biology that is not detected via qualitative visual analyses routinely performed by radiologists [13–18]. Radiomic studies generally serve one of two purposes: classification tasks (*e.g*., discriminating benign *versus* malignant lesions) or prediction tasks (stratification of patients based on the likelihood of occurrence of defined clinical endpoints) [17, 19].

We hereby report the preliminary results of a customized in-house radiomic tool applied to magnetic resonance imaging (MRI) to predict response to upfront CRT in patients affected by locally advanced SCC of the anal canal.

## Methods

### Study population

We retrospectively evaluated pretreatment MRI of 26 consecutive patients (19 females and 7 males, median age of 60.5 years, range 48–83 years) affected by locally advanced (stage III) SCC of the anal canal treated with combination CRT with curative intent at our Institution between July 2012 and June 2022 (Table 1).

**Table 1:** Patients’ demographics and clinical characteristics

Patient ID	Age (years)	Gender	Chemotherapy regimen	Cumulative dose, per cycle (Gy)	Response
PT1	57	F	Carboplatin + Paclitaxel	50.4/59.4	Non-CR
PT2	81	F	Mitomycin + 5-FU	45/59.4	Non-CR
PT3	79	M	Mitomycin + 5-FU	60	Non-CR
PT4	63	F	Mitomycin + 5-FU	45/59.4	Non-CR
PT5	48	M	Capecitabine	41.4/41.4	Non-CR
PT6	58	F	Mitomycin + 5-FU	50.4/59.4	Non-CR
PT7	83	F	Mitomycin + 5-FU	45/50	Non-CR
PT8	59	F	Mitomycin + 5-FU	45/54/60	Non-CR
PT9	65	F	Capecitabine	45/54	Non-CR
PT10	69	F	Mitomycin + 5-FU	45/60	CR
PT11	67	M	Mitomycin + 5-FU	48.6/59.4	CR
PT12	60	F	Mitomycin + 5-FU	45/59.4	CR
PT13	57	M	Mitomycin + 5-FU	45/54/59.4	CR
PT14	52	F	Mitomycin + 5-FU	61.2/46.8	CR
PT15	50	M	Mitomycin + 5-FU	45/54/60	CR
PT16	65	F	Cisplatin + 5-FU	45/54/59.4	CR
PT17	48	M	Mitomycin + 5-FU	45/60	CR
PT18	60	F	Mitomycin + 5-FU	45/59.4	CR
PT19	52	F	Mitomycin + 5-FU	45/54/61.2	CR
PT20	61	M	Mitomycin + 5-FU	45/50.4/59.4	CR
PT21	74	F	Cisplatin + 5-FU	45/60	CR
PT22	60	F	Mitomycin + 5-FU	54	CR
PT23	61	F	Cisplatin + 5-FU	45/50.5/57.6	CR
PT24	72	F	Cisplatin + 5-FU	45/60	CR
PT25	52	F	Cisplatin + 5-FU	45/50/64.7	CR
PT26	75	F	Mitomycin + 5-FU	45/60	CR

*CR* Complete response, *F* Female, *M* Male, *non-CR* Non-complete response, *5-FU* 5-fluorouracil

All patients were staged according to the AJCC-UICC TNM system through a complete diagnostic work-up comprising physical examination, transanal ultrasound, contrast-enhanced whole-body computed tomography (CT), MRI of the lower abdomen and ^18^F-FDG positron emission tomography/CT [20].

The study was approved by the Ethics Committee of the Fondazione IRCCS Istituto Nazionale dei Tumori, Milan, Italy (study INT 90/23).

### Chemotherapy regimens

Eighteen (69%) patients were treated with the combination scheme NIGRO (5-fluorouracil 800–1,000 mg/m^2^/day 1–5 and 29–32; mitomycin C 10 mg/m^2^ 1–29), five patients (19%) received 5-fluorouracil in combination with cisplatin, two (8%) patients received oral capecitabine, and one (4%) patient received carboplatin in combination with paclitaxel (Table 1) [21].

### Radiation therapy protocols

All patients were irradiated at radical doses, reaching 45 Gy on low-risk clinical target volumes followed by a boost of 9–20 Gy on intermediate-risk and high-risk clinical target volumes based on initial disease staging. The dose was delivered via the RapidArc technique−VMAT with conventional fractionation (1.8–2 Gy per fraction) and a median cumulative dose delivered of 59.4 Gy (range 41.4–61.7 Gy) (Table 1).

### Local response

**Fig. 1:**
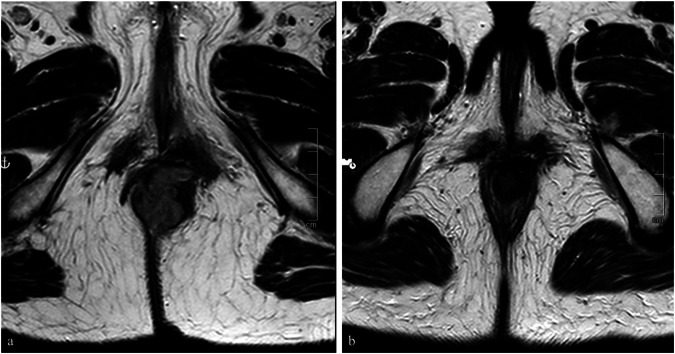
Turbo spin-echo T2-weighted paraxial images demonstrate no residual disease after combination CRT before (**a**) and after treatment (**b**): the patient was classified as a CR case

**Fig. 2:**
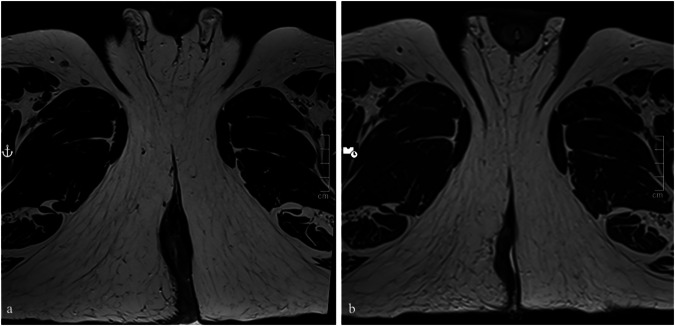
Turbo spin-echo T2-weighted paraxial images demonstrate residual disease after combination CRT before (**a**) and after treatment (**b**): the patient was classified as a non-CR case

Approximately six months after the end of the treatment, patients were classified into two distinct response categories based on the absence or presence of residual disease at MRI and endoscopy: complete response (CR) for 17 patients, 12 females, and 5 males; and non-complete response (non-CR) for 9 patients, 7 females and 2 males (Figs. 1 and 2).

### Image acquisition

Pretreatment imaging was acquired on 1.5-T MRI scanners equipped with phased-array coils for parallel imaging, with the exception of one study acquired on a 1-T scanner (Supplementary Table S1). Acquisition protocols included: T2-weighted turbo spin-echo (TSE) sagittal images, T2-weighted TSE images perpendicular to the long axis of the tumor, T2-weighted TSE paracoronal images for adequate visualization of the sphincter plane, diffusion-weighted imaging with high *b*-values (≥ 800 s/mm^2^) to improve detection of residual disease and large field of view T1-weighted TSE sequences for evaluation of lymph nodes and bone.

Only T2-weighted images from pretreatment MRI were utilized for feature extraction, whereas diffusion-weighted imaging sequences were mostly employed to help evaluate tumor response to CRT.

Acquisition parameters of pretreatment T2-weighted TSE images are detailed in Supplementary Table S1.

Patients’ files in Digital Imaging and Communications in Medicine−DICOM format were full anonymized *via* the dedicated function of the integrated imaging software syngo.via® (Siemens Healthineers, Erlangen, Germany).

### Image segmentation

Regions of interest (ROIs) were manually annotated (slice by slice, whole volume) by two radiologists in training (A.V. and L.V.S.) with dedicated expertise in oncologic imaging and radiomics (≥ 2 years) on T2-weighted TSE para-axial images with the open-source software for image annotation 3DSlicer (https://www.slicer.org/). In case of disagreement, a senior radiologist with ≥ 30 years of experience (D.S.) was consulted to reach a consensus.

### Analysis overview

To maximize reproducibility, feature extraction, model development, and validation were conducted in accordance with the principles of the Image Biomarker Standardization Initiative [22].

**Fig. 3:**
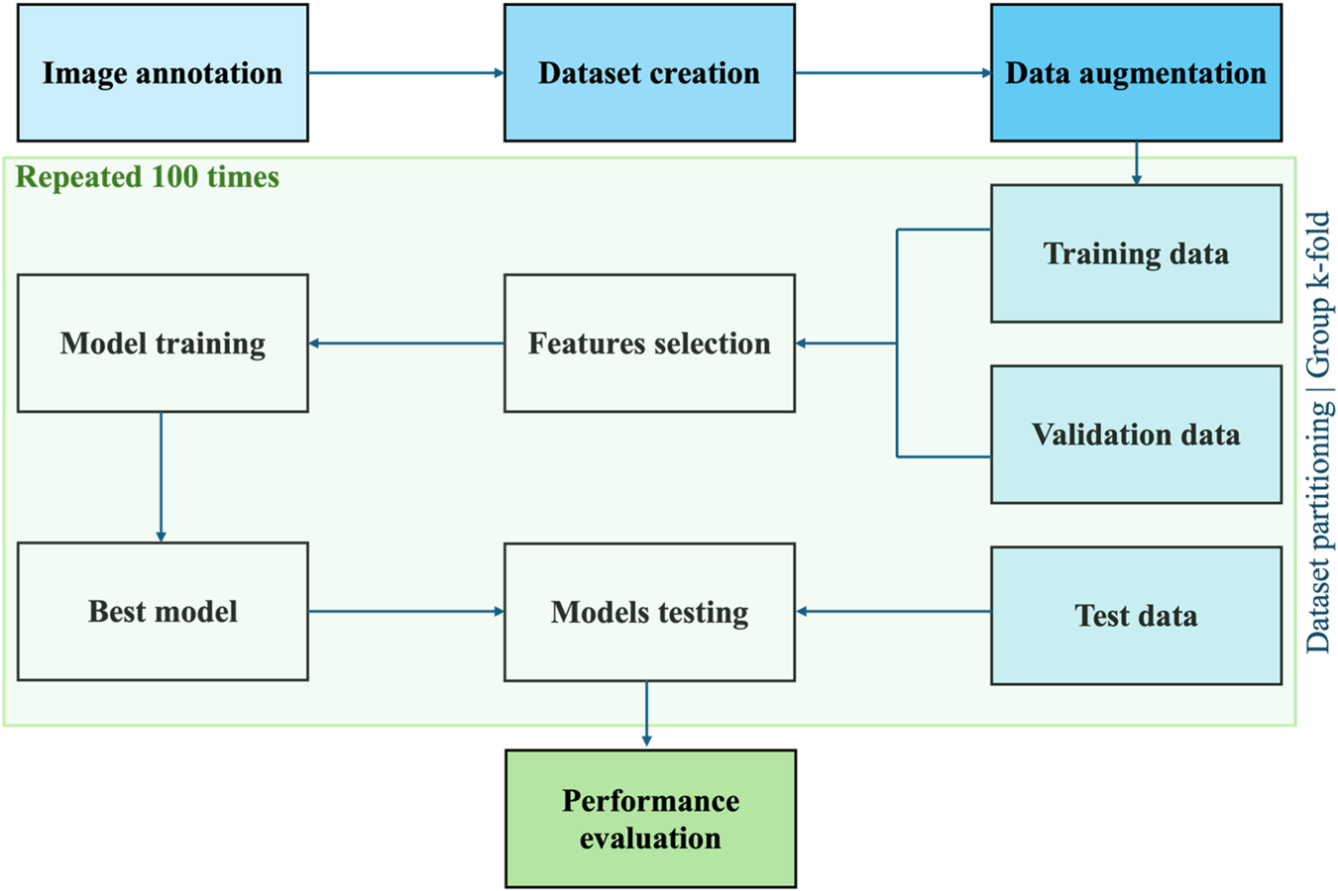
Analysis of workflow

The methodological robustness of our study has been assessed with the METhodological RadiomICs Score (METRICS) [23], achieving a total score of 81.5% (“excellent”). Results are detailed in Supplementary Fig. S1. The analysis workflow is depicted in Fig. 3.

#### Data augmentation

To enhance the statistical power of our analysis, we implemented a customized data augmentation tool to artificially increase the size of our dataset via the geometric manipulation of native images. Data augmentation was achieved using perturbation techniques, which have been proven to enhance the generalization of machine learning models more effectively than tabular data augmentation methods such as synthetic minority over-sampling techniques or adaptive synthetic sampling. This approach is particularly advantageous in texture analysis, where neighboring pixel relationships are directionally dependent and require a more nuanced approach to maintain spatial and contextual integrity [24, 25]. An augmentation factor of 9 was applied to the CR group, while the non-CR group was augmented by a 17-fold change. As a result, a total of 266 instances was obtained, ensuring a balanced representation with 133 data points for each category. To achieve this, we generated random three-dimensional roto-translations for each image and its corresponding label. Specifically, three Euler angles (within ± 5 degrees for left-right and anterior-posterior, ± 15 degrees for inferior-superior) and three translation values (within ± 5 mm) were extracted to create a roto-translation matrix [26, 27]. This matrix was applied to both the image and label, transforming them accordingly. Scaling was avoided to preserve image realism [15, 25].

#### Features extraction

For each individual patient, regardless of whether their data had been augmented or not, we performed image normalization. More specifically, each image was scaled by dividing its pixels’ intensities by its own maximum value. Subsequently, a set of 132 three-dimensional radiomic features was extracted using a customized extraction tool [28]. Images were resampled using a B-spline interpolator available in ITK 4.13 for smooth intensity preservation, while ROIs were resampled with a nearest-neighbor approach to maintain discrete labels and avoid boundary inaccuracies.

The extracted radiomic features pertained to the following categories:

- Shape and size (SS) features (*n* = 61), which describe the geometric properties of ROIs, providing information on the tumor’s shape and morphology;
- Intensity-based features, also known as first-order features (*n* = 37), which describe the spatial distribution of signal intensities within the ROI;

- Second-order features, also known as “texture” features, which describe spatial complexity and relationships between signal intensities of neighboring voxels:
  - Gray level co-occurrence matrix (GLCM; *n* = 23) analyses the signal intensities of pairs of pixels located at a given distance and calculates how often pairs of pixels with a specific value occur in the ROI;
  - Gray level run length matrix (GLRLM; *n* = 11) quantifies gray level runs, namely the number of consecutive pixels with equal intensity.

The specific parameters used for feature extraction were chosen based on established guidelines [22].

Notably, the number of intensity bins used for histogram discretization was set to 32 to ensure sufficient sensitivity to subtle variations in intensity within the ROI. Additionally, the minimum and maximum values of the histogram were determined by the minimum and maximum intensity values within the image itself, ensuring that the extracted features were highly sensitive to subtle variations in T2-weighted images.

The radiomics features were extracted using the feature extractor developed for BD2Decide (bd2decide.eu) [15, 16, 19, 28–30].

### Features selection

First, radiomic features were categorized into six distinct groups: first-order statistics (FOS); FOS Histogram; FOS Signal; GLCM; GLRM; and SS. Then, the Gini impurity index was employed to select the five most informative features from each subset, thus creating six additional ones (“_subset5”). Lastly, two additional subsets, one containing all the 132 extracted features (“All”) and the other containing the five most informative features across all subsets combined (“All_subset5”), were created. As a result, a total of 14 subsets, each representing a different grouping of features, was obtained (Table 2).

**Table 2:** Performances of the trained models for each of the 14 subsets of features

	Sensitivity	Specificity	Accuracy	AUC
All	0.661 ± 0.183	0.517 ± 0.403	0.599 ± 0.169	0.589 ± 0.165
All_subset5	0.894 ± 0.122	0.794 ± 0.237	0.824 ± 0.154	0.844 ± 0.128
FOS	0.750 ± 0.186	0.317 ± 0.207	0.523 ± 0.099	0.533 ± 0.116
FOS_subset5	0.861 ± 0.134	0.617 ± 0.314	0.721 ± 0.148	0.739 ± 0.128
FOS Histogram	0.767 ± 0.122	0.222 ± 0.230	0.486 ± 0.131	0.494 ± 0.117
FOS Histogram_subset5	0.756 ± 0.224	0.350 ± 0.300	0.548 ± 0.165	0.553 ± 0.190
FOS Signal	0.800 ± 0.163	0.672 ± 0.266	0.697 ± 0.102	0.736 ± 0.073
FOS Signal_subset5	0.900 ± 0.133	0.733 ± 0.143	0.809 ± 0.066	0.817 ± 0.068
GLCM	0.900 ± 0.122	0.750 ± 0.211	0.799 ± 0.056	0.825 ± 0.049
GLCM_subset5	0.900 ± 0.122	0.833 ± 0.175	0.846 ± 0.064	0.867 ± 0.055
GLRLM	0.656 ± 0.199	0.406 ± 0.270	0.541 ± 0.194	0.531 ± 0.231
GLRLM_subset5	0.650 ± 0.200	0.433 ± 0.347	0.523 ± 0.132	0.542 ± 0.102
SS	0.550 ± 0.277	0.333 ± 0.380	0.433 ± 0.260	0.442 ± 0.268
SS_subset5	0.733 ± 0.276	0.350 ± 0.436	0.539 ± 0.193	0.542 ± 0.195

Performances across the 100 models derived from different data splitting are reported as average ± standard deviation. The “All” subset represents the model trained using all 132 extracted features. Each row reports the performances of the model trained with the corresponding class of radiomic features. “_subset5” identifies the models trained with the top-performing features from each individual class

*AUC* Area under the receiver operating characteristics curve, *FOS* First-order features, *GLCM* Gray-level co-occurrence matrix, *GLRLM* Gray-level run-length matrix

The whole process, including the identification of the five most informative features for each subset, was repeated 100 times. During each iteration, we randomly sampled 80% of the dataset to ensure the stability and generalizability of the results. This repetition process helped to account for potential biases and improved the overall reliability of the identified informative features.

### Machine learning

In consideration of the suboptimal performances often displayed by conventional single models when operating with small datasets, we employed a comprehensive machine learning approach leveraging a customized multi-model strategy first described by An et al [31, 32]. This approach entails training a model multiple times using in each instance an independent combination of training, validation, and testing split, thus minimizing overfitting while at the same time ensuring robustness and reproducibility [31]. For each of the 16 subsets of features, we trained 100 independent naive Bayesian models. This involved generating 100 unique combinations of training, validation, and testing sets using a 60%/20%/20% split of the 266 datasets (Fig. 3).

We ensured a balanced representation by stratifying the datasets based on the same amount of data in the two classes (CR and non-CR). Each of the 100 models underwent a rigorous 10-fold cross-validation process.

This ensured that no model was exposed to its corresponding testing set during training, bolstering the reliability of the results. This approach resulted in the training of a total of 16,000 naive Bayesian models (16 subsets × 100 naive Bayesian models × 10 cross-validations). For each model, the input was represented by the extracted radiomic features and the output was the classification of CR *versus* non-CR.

To select the most effective model for each subset, we evaluated the prediction accuracy of all 100 models using the 10-fold cross-validation results. Only the model with the highest prediction accuracy was then evaluated on its corresponding testing dataset. To ensure the integrity and validity of our machine learning analysis, we actively addressed the potential for data leakage.

In the context of data augmentation, where synthetic instances are generated to augment the training dataset, it is crucial to avoid situations where augmented data for a specific patient leaks into its corresponding testing set. Such leakage can artificially inflate the model’s performance and lead to inaccurate results.

To prevent this issue, we forced the split function to follow the rule that augmented data for a patient could only be included in the same set (training, validation, or testing) as the original data of that patient. This approach ensured that the model was not exposed to any information about the augmented data during training or validation, thereby avoiding any potential leakage and preserving the integrity of our analysis.

We evaluated the diagnostic performance of each subset of features with a Wilcoxon rank-sum test (significant at *p*-value < 0.05).

## Results

Multiple subsets of features were utilized for prediction modeling, including first-order features, GLCM features, GLRLM) features, and SS features. Our model predicted tumor response to CRT with statistically significant accuracy, highlighting several subsets of features—including GLCM_subset 5, FOS Signal_subset 5, and All_subset 5—endowed with satisfactory performances (accuracy ≥ 0.8) (Table 2).

**Fig. 4:**
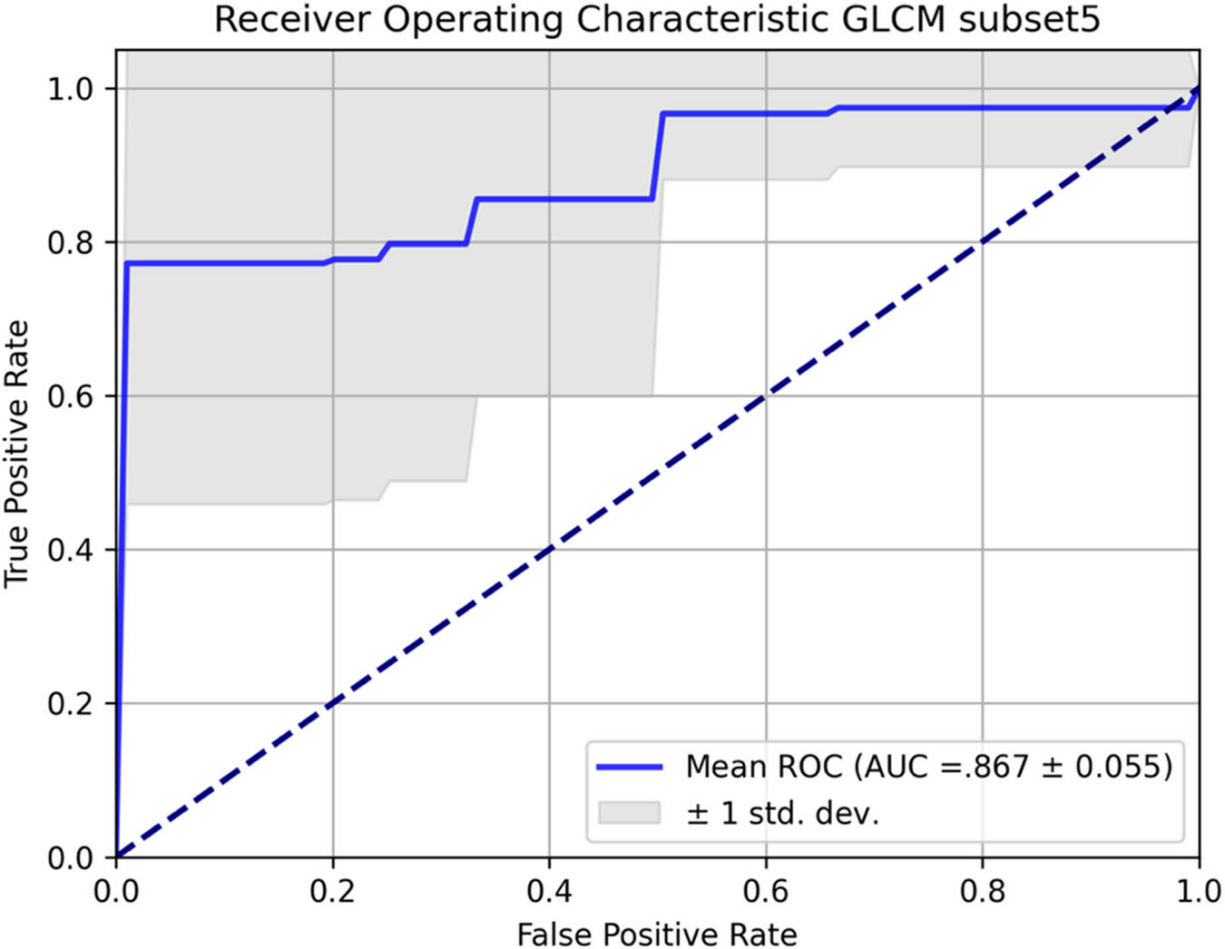
Receiver operating characteristic (ROC) curve of the best-performing subset of features (GLCM_subset5). GLCM, Gray level co-occurrence matrix

**Fig. 5:**
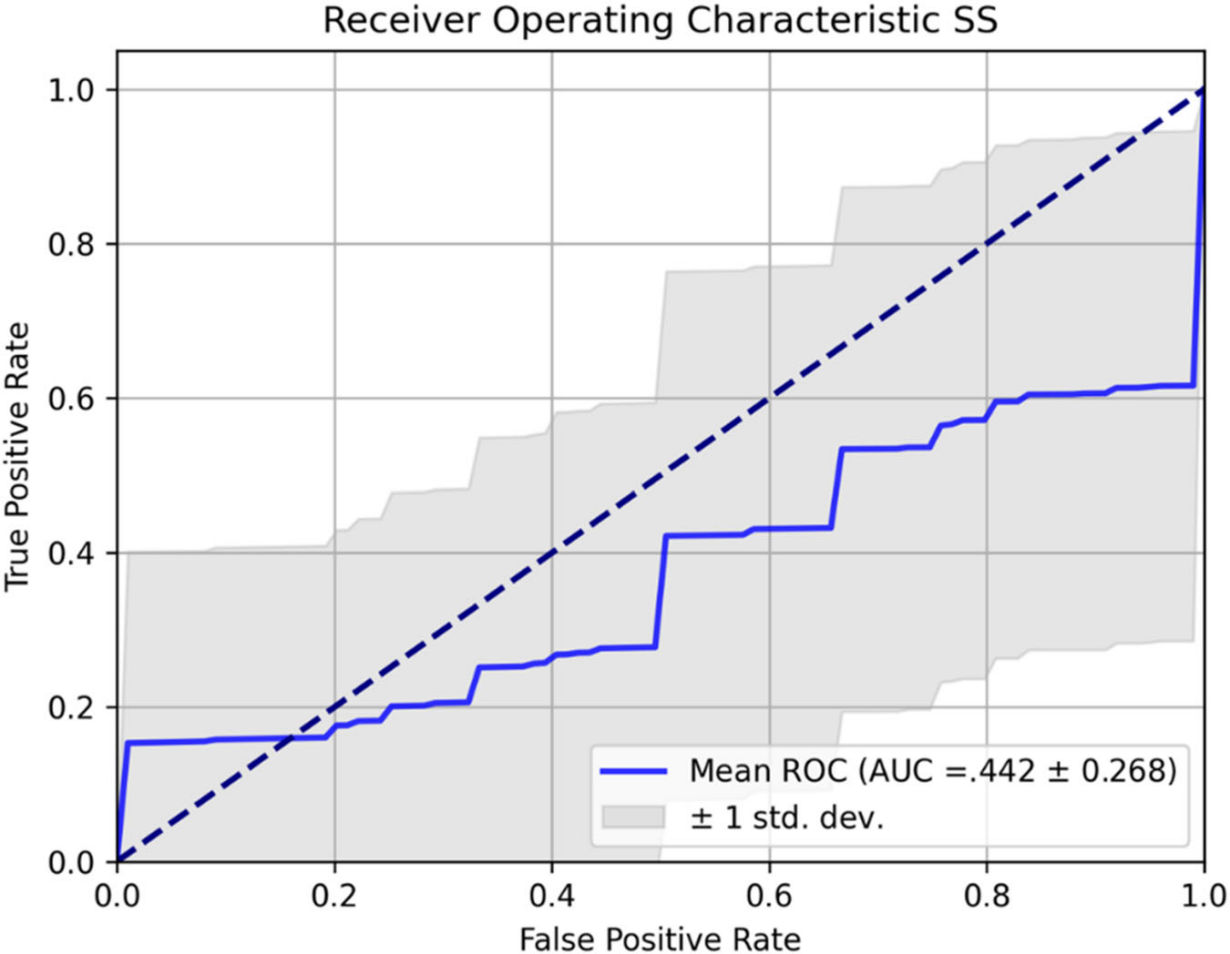
Receiver operating characteristic curve (ROC) of the worst-performing subset of features. SS, Shape and size

However, performances varied significantly across different models. The receiver operating characteristic curves for the best (GLCM_subset5) and worst (SS) performing features are reported in Figs. 4 and 5.

The models trained using the GLCM features ranked first, achieving the best performances across all metrics of the confusion matrix, with an accuracy of 0.846 ± 0.064, sensitivity of 0.900 ± 0.122, and specificity of 0.833 ± 0.175, with an area under the curve (AUC) of 0.867 ± 0.055. The second-best performing subset was derived from FOS Signal features, which provided values of 0.809 ± 0.066, 0.900 ± 0.133, 0.733 ± 14.3, and 0.817 ± 0.068. On the other hand, FOS Histogram features demonstrated significantly weaker performances, with values of 0.548 ± 0.165, 0.756 ± 0.224, 0.350 ± 0.300, and 0.553 ± 0.190.

Models trained with the GLRLM subset performed poorly compared to GLCM and FOS features, achieving an accuracy of 0.523 ± 0.132, with a sensitivity of 0.650 ± 0.200 and a specificity of 0.433 ± 0.347, resulting in an AUC of 0.542 ± 0.102; SS features also demonstrated unsatisfactory predictive power, with an accuracy of 0.539 ± 0.193, a sensitivity of 0.733 ± 0.276, and a specificity of 0.350 ± 0.436. The AUC for this subset was only 0.542 ± 0.195, indicating that geometric properties of the tumor, such as size and shape, did not provide sufficient discriminatory information to classify patients’ response to CRT based on pretreatment imaging.

**Fig. 6:**
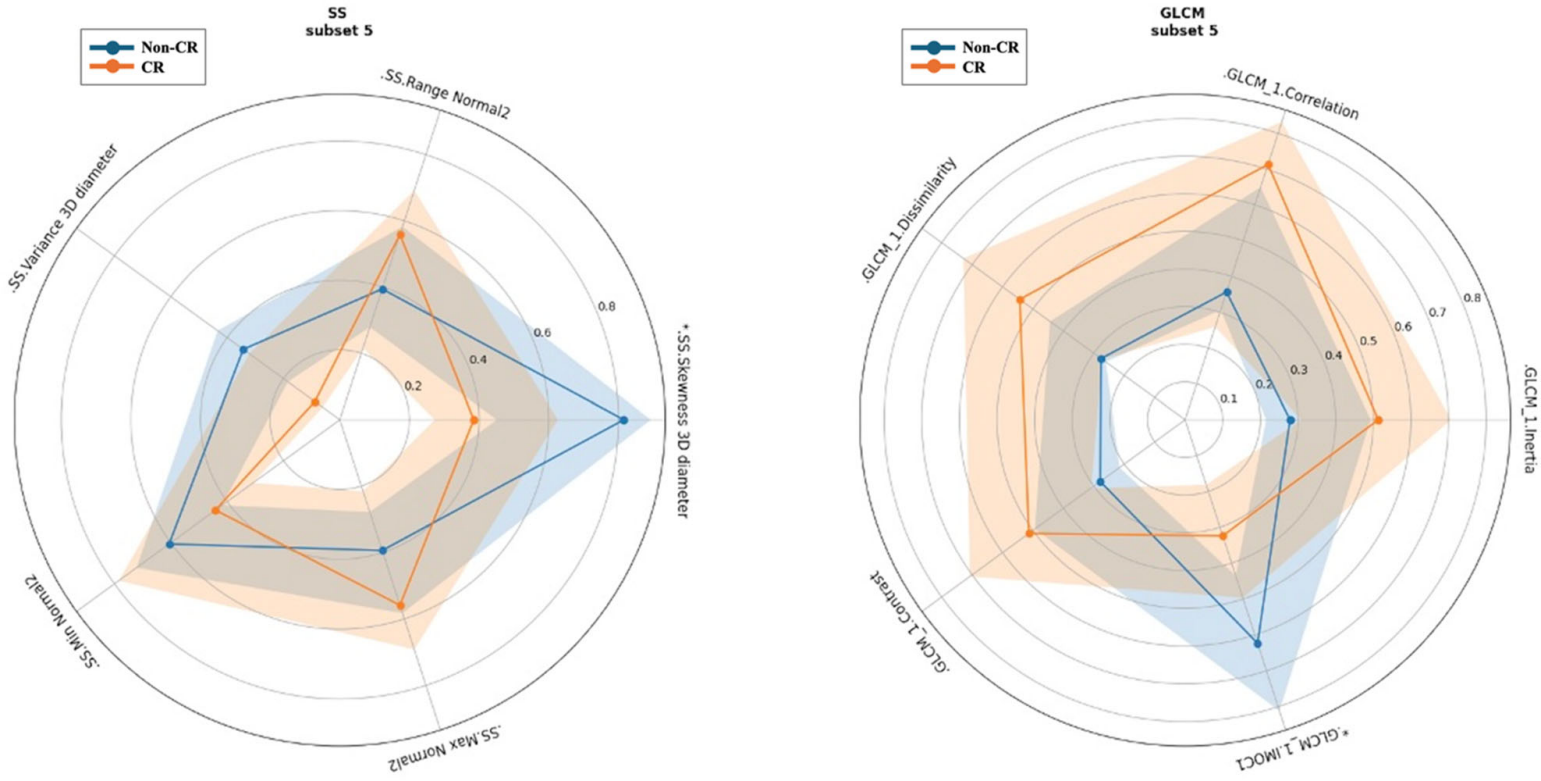
Radar plots showing the normalized values of the five most correlated radiomic features in the worst-performing subsets (left panel) and best-performing subsets (right panel). The bold line represents the average value across all subjects/patients, whereas the shaded area includes from the 25th to the 75th percentile of the distribution of the metrics/features. The asterisk (*) indicates a *p*-value < 0.01 in differentiating non-CR from CR patients

Overall, first-order and GLCM features reported a significantly more homogeneous distribution of voxel intensities, and therefore greater tissue homogeneity, in patients with incomplete responses to CRT (Fig. 6).

These results highlight the importance of texture-based radiomic features, particularly those derived from GLCM, in predicting treatment response in patients with SCC of the anal canal.

## Discussion

Carcinomas of the anal canal are rare and pose peculiar technical challenges due to their distinctive anatomical constraints, which make it particularly complex to achieve an acceptable compromise between radicality of treatment and quality of life [33–35].

Intratumoral heterogeneity is now widely accepted among the drivers of therapy resistance, either primary or acquired, and its comprehension is therefore of paramount importance for the development of effective therapies [36–41]. Single-cell technologies currently represent the gold standard for dissecting intratumoral heterogeneity [42–44]. However, the ongoing revolution of quantitative imaging and radiomics hints that bioimages may also be utilized to this end [45–49]. We have developed a customized radiomic algorithm to deconvolute tissue complexity and leverage differences in radiomic texture at pretreatment imaging to predict tumor response to upfront CRT.

We report that first-order and GLCM features, which describe the spatial distribution and complexity of voxel intensities within the ROI, demonstrated a more homogeneous tumor composition in patients with non-CR. For models trained GLCM features, the accuracy was 0.846 ± 0.064, sensitivity 0.900 ± 0.122, specificity 0.833 ± 0.175, and the area under receiver operating characteristics curve 0.867 ± 0.055. This performance may be explained by the existence of a dominant neoplastic clone with a low mitotic index, therefore lower rates of cystic degeneration and necrosis, which responds less dramatically to chemotherapy and radiotherapy, both of them being more likely to be effective on rapidly proliferating cellular populations [50–52]. Conversely, limited predictive power was shown by SS features, demonstrating that geometric properties of the lesions did not discriminate efficiently CR patients from non-CR patients. Notably, this diverges from observations in other malignancies, in which tumor morphology was proved to be predictive of disease aggressiveness and clinical outcomes [53]. These results should be considered preliminary because lacking external validation, yet—in our opinion—pave the way for treatment optimization for patients affected by stage III SCC of the anal canal.

A particular aspect of our study population was the imbalance between CR and non-CR patients, which reflects real-life proportions. We addressed this issue with a multi-layered strategy. First, we employed an *ad hoc* data augmentation tool based on the geometric manipulation of images to differentially increase each subset of patients and reach an almost perfect balance between the two response categories. Augmented data was included in both training and testing datasets. While this strategy may seem unconventional and potentially raise some concerns, it is thought to increase model robustness by simulating real-world variability [54]. Secondly, we implemented a rigorous method to select top-performing features and filtered out all toxic (non-informative or detrimental) contexts. Feature stability and correlation will be further addressed in future work. Lastly, we did not employ a single model of machine learning, as usually done in this kind of analysis, but a customized multi-model classifier with enhanced discriminative performances. Additional robustness was achieved thanks to the 100-fold iteration factor employed at each step of the analysis.

This study has limitations. First, it is retrospective in design, and, as such, susceptible to selection bias. To minimize unwanted technical variability, we included all consecutive patients affected by stage III SCC of the anal canal treated at our institution from 2012 to 2022. Second, the number of patients included is limited. However, one should consider the relative rarity of the disease. To the best of our knowledge, this represents one of the largest casuistries reported in the literature for this specific clinical scenario. The monocentric design of this study accounts for the extensive homogeneity in patients’ clinical management and follow-up. We have persuaded this consistency significantly improved the robustness and reproducibility of our model, a matter of particular concern in radiomics.

In conclusion, we showed that a radiomic tool based on manual segmentation of T2-weighted pretreatment images accurately predicts tumor response to CRT in patients with stage III SCC of the anal canal, highlighting a more homogeneous tissue composition in poor responders. We provided an original contribution in a clinical scenario where radiomics has had limited application so far. In addition, our results pave the way for the development of innovative tools to stratify patients at diagnosis in a clinical context in which reproducible clinical and molecular prognostic tools are scarce. External validation on larger cohorts of patients and integration with other “-omics” sciences [55, 56] will be needed to bridge the gap between these preliminary, mostly computational results, and clinical practice.

## Supplementary information

**Additional file 1: Supplementary Table S1.** Acquisition parameters of pre-treatment T2-weighted TSE sequences utilized to extract radiomic features. **Supplementary Fig. S1.** Results of the METhodological RadiomICs Score METRICS), a quality scoring tool for radiomics research.

## Data Availability

All steps of the analysis are detailed in the body of the article. Results are reported in Table 2 and Figs. 4–6. All additional information, including raw data, can be shared upon reasonable request to the corresponding author.

## Abbreviations

AUC: Area under the receiver operating characteristics curve
CR: Complete response
CRT: Chemoradiotherapy
CT: Computed tomography
GLCM: Gray-level co-occurrence matrix
GLRLM: Gray-level run-length matrix
MRI: Magnetic resonance imaging
Non-CR: Non-complete response
ROI: Region of interest
SCC: Squamous cell carcinoma
SS: Shape and size
TSE: Turbo spin-echo
