# Supplementary material for: Radiomics to predict tumor response to combination chemoradiotherapy in squamous cell carcinoma of the anal canal: a preliminary investigation
Source: Eur Radiol Exp. 2025 Mar 22;9:35. doi: 10.1186/s41747-025-00559-0 (PMC11929663; doi:10.1186/s41747-025-00559-0)
Supplement: Supplementary file 1 — Additional file 1: Supplementary Table S1. Acquisition parameters of pre-treatment T2-weighted TSE sequences utilized to extract radiomic features. Supplementary Fig. S1. Results of the METhodological RadiomICs Score METRICS), a quality scoring tool for radiomics research. [file 41747_2025_559_MOESM1_ESM.pdf]

Radiomics to predict tumor response to combination chemoradiotherapy in squamous cell carcinoma of the anal canal: a preliminary investigation

ELECTRONIC SUPPLEMENTARY MATERIAL

Supplementary Table S1 Acquisition parameters of pre-treatment T2-weighted TSE sequences utilized to extract radiomic features

Patient ID	Magnetic field strength T)	Scanner	Slice thickness mm)	Field of view mm)	Spacing between slices mm)	Time of echo ms)	Time of repetition ms)	Flip angle °)	Echo train length	Acquisition matrix	Pixel bandwith	Number of averages	Phase encoding steps
PT1	1,5	Siemens Avanto	2	200x200	2,4	86	3740	180	15	256x192	200	3	391
PT2	1,5	Siemens Aera	3	400x400	4,05	104	7290	129	30	384x288	200	1	315
PT3	1,5	Siemens Avanto	3	170x170	3,6	111	5260	150	13	256x256	121	1	520
PT4	1,5	Philips Ingenia	3,5	240x240	3,85	110	3707,46	90	16	320x320	312	2	320
PT5	1,5	Philips Ingenia	3	200x200	3,3	110	4657,47	90	13	332x163	109	1	163
PT6	1,5	Philips Ingenia	5	380 x380	5,5	80	434,98	90	71	272x237	676	1	237
PT7	1,5	Philips Ingenia	5	280x280	6,5	100	3464,96	90	18	176x130	188	1	130
PT8	1	Philips Panorama	4	260x260	4,4	100	3864,93	90	16	172x160	173	1	160
PT9	1,5	Siemens Avanto	3	250x250	4,05	94	6810	150	18	320x240	130	2	360
PT10	1,5	Siemens Avanto	3	170x170	3,6	111	5260	150	13	256x256	121	1	520
PT11	1,5	Philips Achieva	3,5	240x240	3,85	90	3000	90	20	320x312	346	1	337
PT12	1,5	Siemens Avanto	2	200x200	2,4	86	3740	180	15	256x192	200	3	391
PT13	1,5	Siemens Avanto	2	200x200	2.4	86	3740	177	15	256x192	200	3	391
PT14	1,5	Siemens Avanto	3	250x190	3,6	148	4150	150	21	320x216	130	3	336
PT15	1,5	Philips Ingenia	5	350x350	6	100	2453,79	90	45	352x45	564	1	352
PT16	1,5	Philips Ingenia	3,5	150x150	3,85	110	5141,06	90	32	232x230	364	2	230
PT17	1,5	Siemens Avanto	2	200x200	2,4	86	3740	180	15	256x192	200	3	391
PT18	1,5	Philips Ingenia	3	180x180	3,3	100	3708,21	90	20	240x215	220	3	215
PT19	1,5	Siemens Aera	2,5	160x160	2,875	164	14240	120	30	192x192	150	1	192
PT20	1,5	Siemens Aera	6	380x300	7,2	90	1100	180	80	320x182	710	1	118
PT21	1,5	Siemens Avanto	3	170x170	3,6	111	5260	150	13	256x256	121	1	520
PT22	1,5	Siemens Avanto	3	170x170	3,6	110	4380	150	13	256x256	121	1	390
PT23	1,5	Siemens Aera	3	400x460	3,3	104	7390	129	30	384x243	200	1	270
PT24	1,5	Philips Achieva	3	200x200	3,3	120	4000	90	23	248x215	235	3	215
PT25	1,5	Siemens Aera	3	380x460	3,3	104	9360	129	30	384x243	200	1	270
PT26	1,5	Siemens Avanto	3	260x200	3,6	151	4000	150	21	320x216	130	3	379

Supplementary Fig. S1 Results of the METhodological RadiomICs Score METRICS), a quality scoring tool for radiomics research

METRICS Tool v1.0

Please fill out all conditions first for relevant sections and then all active items to calculate METRICS score.

Please note that default option is "No".

? Stands for explanation of items and conditions.

C Stands for conditional items or sections.

Items/Conditions	Definitions	Weights	Options
**Study Design**			
Item#1	? Adherence to radiomics and/or machine learning-specific checklists or guidelines	0.0368	● Yes ○ No
Item#2	? Eligibility criteria that describe a representative study population	0.0735	● Yes ○ No
Item#3	? High-quality reference standard with a clear definition	0.0919	● Yes ○ No
**Imaging Data**			
Item#4	? Multi-center	0.0438	○ Yes ● No
Item#5	? Clinical translatability of the imaging data source for radiomics analysis	0.0292	● Yes ○ No
Item#6	? Imaging protocol with acquisition parameters	0.0438	● Yes ○ No
Item#7	? The interval between imaging used and reference standard	0.0292	● Yes ○ No
**Segmentation** C			
Condition#1	? Does the study include segmentation?		● Yes ○ No
Condition#2	? Does the study include fully automated segmentation?		○ Yes ● No
Item#8	? Transparent description of segmentation methodology	0.0337	● Yes ○ No
Item#9	? Formal evaluation of fully automated segmentation C	0.0225	○ Yes ○ No
Item#10	? Test set segmentation masks produced by a single reader or automated tool	0.0112	○ Yes ● No
**Image Processing and Feature Extraction**			
Condition#3	? Does the study include hand-crafted feature extraction?		● Yes ○ No
Item#11	? Appropriate use of image preprocessing techniques with transparent description	0.0622	● Yes ○ No
Item#12	? Use of standardized feature extraction software C	0.0311	● Yes ○ No
Item#13	? Transparent reporting of feature extraction parameters, otherwise providing a default configuration statement	0.0415	● Yes ○ No
**Feature Processing**			
Condition#4	? Does the study include tabular data?		● Yes ○ No
Condition#5	? Does the study include end-to-end deep learning?		○ Yes ● No
Item#14	? Removal of non-robust features C	0.0200	● Yes ○ No
Item#15	? Removal of redundant features C	0.0200	● Yes ○ No
Item#16	? Appropriateness of dimensionality compared to data size C	0.0300	● Yes ○ No
Item#17	? Robustness assessment of end-to-end deep learning pipelines C	0.0200	○ Yes ○ No
**Preparation for Modeling**			
Item#18	? Proper data partitioning process	0.0599	● Yes ○ No
Item#19	? Handling of confounding factors	0.0300	● Yes ○ No
**Metrics and Comparison**			
Item#20	? Use of appropriate performance evaluation metrics for task	0.0352	● Yes ○ No
Item#21	? Consideration of uncertainty	0.0234	● Yes ○ No
Item#22	? Calibration assessment	0.0176	● Yes ○ No
Item#23	? Use of uni-parametric imaging or proof of its inferiority	0.0117	● Yes ○ No

Item#24	?	Comparison with a non-radiomic approach or proof of added clinical value	0.0293	○ Yes ● No
Item#25	?	Comparison with simple or classical statistical models	0.0176	○ Yes ● No
**Testing**				
Item#26	?	Internal testing	0.0375	● Yes ○ No
Item#27	?	External testing	0.0749	○ Yes ● No
**Open Science**				
Item#28	?	Data availability	0.0075	● Yes ○ No
Item#29	?	Code availability	0.0075	● Yes ○ No
Item#30	?	Model availability	0.0075	● Yes ○ No
			**Total METRICS score:**	**81.5%**
			? **Quality category:**	**Excellent**
			? **Publication ID:**	
